# Novel interactomics approach identifies ABCA1 as direct target of evodiamine, which increases macrophage cholesterol efflux

**DOI:** 10.1038/s41598-018-29281-1

**Published:** 2018-07-23

**Authors:** Limei Wang, Pierre Eftekhari, Daniel Schachner, Irena D. Ignatova, Veronika Palme, Nicole Schilcher, Angela Ladurner, Elke H. Heiss, Herbert Stangl, Verena M. Dirsch, Atanas G. Atanasov

**Affiliations:** 1 Department of Pharmacognosy, University of Vienna, Vienna, Austria; 2 Department of Pharmacology, School of Pharmacy, Qingdao University, Qingdao, 266021 Shandong Province China; 3 Inoviem Scientific, Strasbourg, France; 4 Department of Pharmacology, University of Virginia Health System, Charlottesville, VA USA; 5 Institute of Medical Chemistry, Center for Pathobiochemistry and Genetics, Medical University of Vienna, Vienna, Austria; 6 Institute of Genetics and Animal Breeding of the Polish Academy of Sciences, 05-552 Jastrzebiec, Poland

## Abstract

Evodiamine, a bioactive alkaloid from the fruits of the traditional Chinese medicine *Evodia rutaecarpa* (Juss.) Benth. (Fructus Evodiae, Wuzhuyu), recently gained attention as a dietary supplement for weight loss and optimization of lipid metabolism. In light of its use by patients and consumers, there is an urgent need to elucidate the molecular targets affected by this natural product. Using a novel interactomics approach, the Nematic Protein Organisation Technique (NPOT), we report the identification of ATP-binding cassette transporter A1 (ABCA1), a key membrane transporter contributing to cholesterol efflux (ChE), as a direct binding target of evodiamine. The binding of evodiamine to ABCA1 is confirmed by surface plasmon resonance (SPR) experiments. Examining the functional consequences of ABCA1 binding reveals that evodiamine treatment results in increased ABCA1 stability, elevated cellular ABCA1 protein levels, and ultimately increased ChE from THP-1-derived human macrophages. The protein levels of other relevant cholesterol transporters, ABCG1 and SR-B1, remain unaffected in the presence of evodiamine, and the ABCA1 mRNA level is also not altered.

## Introduction

This study demonstrates the potency of NPOT as a novel interactomics approach to identify molecular targets of small molecules. Furthermore, the identified bioactivity makes evodiamine a good candidate to be further explored for therapeutic or preventive application in the context of cholesterol metabolism.

**Figure 1:**
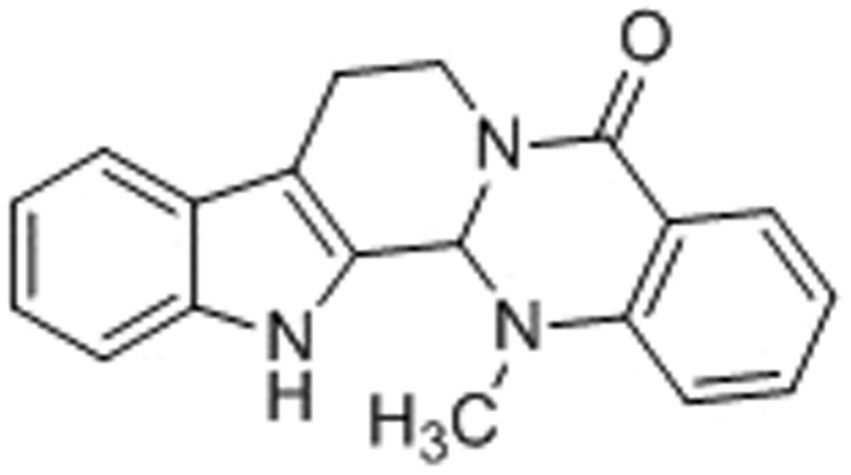
Chemical structure of evodiamine.

Evodiamine **(**Fig. 1**)** is a indoloquinazoline alkaloid isolated from the fruits of *Evodia rutaecarpa* (Juss.) Benth. (Fructus Evodiae, Wuzhuyu)^1,2^, a prominent traditional Chinese medicine, widely used to treat headache, abnormal cold, abdominal pain, diarrhea, hypertension, and mouth ulcers, among others^3^. Growing evidence demonstrates that preparations of *E. rutaecarpa* exhibit beneficial cardiovascular effects, such as lowering blood pressure in dogs, inhibiting platelet aggregation in rabbits, protecting rats against acute myocardial ischemia, and vasodilating properties *in vitro*^4–6^. Since evodiamine is an abundant alkaloid in *E. rutaecarpa*, studies addressing its bioactivities are gaining increasing attention. Of particular interest is its potential as weight-lowering food supplement. Several studies have demonstrated reduced fat accumulation and body weight after evodiamine supplementation in mice and rats^7–9^. In a randomized double-blind clinical trial, the body mass index (kg/m^2^) in premenopausal women was significantly reduced after administration of an *Evodia* extract in capsules (evodiamine 6.75 mg, rutaecarpine 0.66 mg)^10^. However, another randomized controlled trial found that acute ingestion of 500 mg evodiamine at rest followed by 30 min of moderate exercise was ineffective in terms of thermogenesis induction and enhanced fat oxidation in men^11^. As an independent predictor of cardiovascular disease (CVD), obesity (especially visceral obesity) significantly increases the risk of CVD^12,13^. A recent study indicated that evodiamine may protect against CVD by lowering plasma triglyceride (TG), total cholesterol (TC), and low density lipoprotein-cholesterol (LDL-C) level and by raising HDL-cholesterol (HDL-C) level in hyperlipidemic mice^14^. Importantly, many commercial nutritional supplements containing evodiamine are recently marketed to be targeting a range of indications related to improvement of metabolism and weight reduction, and can be readily purchased by patients and consumers from online shops.

Taken together, envisaging nutritional or pharmacological applications in the context of obesity and hyperlipidemia, it is of high interest to know the molecular targets affected by evodiamine.

Nematic protein organisation technique (NPOT) is a label free proprietary technology by INOVIEM Scientific dedicated to the isolation and identification of specific macromolecular scaffolds implemented in basic conditions or in pathological situations directly from human tissues. This proprietary technology is based on the Kirkwood-Buff molecular crowding and aggregation theory^15,16^. It enables the formation and label-free identification of macromolecular complexes involved in physiological or pathological processes, including the analysis of protein-protein and protein-ligand interactions in cells or human tissue, without disrupting the native molecular conformation.

In this work we have aimed at demonstrating the potency of NPOT as a novel approach to identify direct molecular targets of small molecules. Using NPOT and subsequent surface plasmon resonance (SPR) experiments to confirm obtained results, we show that evodiamine directly binds to the ChE transporter ABCA1, resulting in extended ABCA1 protein half-life, and ultimately increased ChE mediated by this transporter. These findings validate the use of NPOT as interactomics approach in the search of molecular targets of small molecules, and shed new light on the molecular mechanism by which the popular nutritional supplement evodiamine affects cholesterol metabolism.

## Results

### ABCA1 is a direct binding target of evodiamine

**Figure 2:**
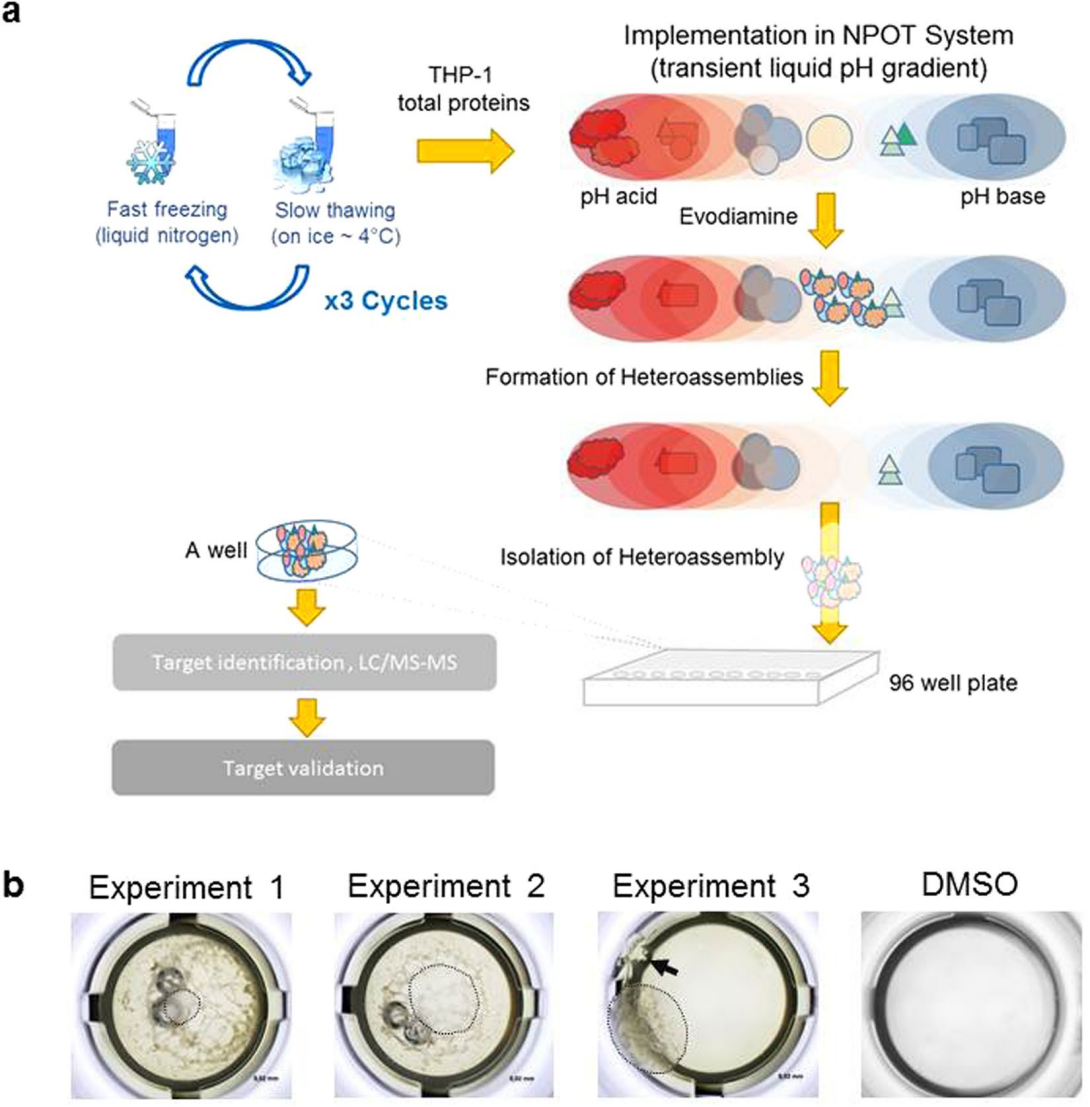
Nematic protein organisation technique (NPOT) for interactome analysis of evodiamine. (**a**) The NPOT was performed under laminar flow and sterile conditions at 4 °C. 1 µM of evodiamine was mixed separately with 1 µg of total THP-1 extract and then subjected to NPOT heteroassemblies isolation. Experiments were performed three times independently. After the NPOT isolation step, the heteroassemblies were allowed to form overnight, and captured in 96 wells microtiter plates in the form of a droplet. Finally, the heteroassemblies are isolated and identified by mass spectroscopy directly in liquid. **(b)** Capture of heteroassemblies in a droplet using 96 well microtiter plate. The floating heteroassemblies are circled with dotted line in three independent experiments. Non interacting proteins are precipitated to the bottom of the well in experiment 1 and 2. The latter is visible in the third experiment on the wall of the well as pointed with the red arrow. The latter is normally due to the vibration induced by incubator ventilation. In the absence of evodiamine no heteroassambly is formed (DMSO), as seen in the last picture on the right.

To identify direct targets of evodiamine, we performed a NPOT interactome analysis of the compound in THP-1-derived human macrophages (Fig. 2). Among the identified targets of evodiamine was the cholesterol transporter ABCA1 **(**Supplementary Table 1**)**. ABCA1 is a key membrane ChE transporter, which is considered to counteract development of atherosclerosis by promoting cholesterol export from macrophages, overall contributing to reverse cholesterol transport (RCT), a process that eliminates cholesterol from the body^17,18^. In addition, four further target proteins have been identified that are reported to be directly involved in atherosclerosis, namely complement C3, complement C4-A, complement component C9, and ceruloplasmin^19–22^. Among the other identified proteins, four play a role in blood coagulation, namely coagulation factor XIII A chain, kininogen-1, fibrinogen beta chain, and fibrinogen gamma chain^23^. Retinal dehydrogenase 1, retinol-binding protein 1, and regucalcin are implicated in cholesterol and fat metabolism as well as in hyperlipidemia and diabetes. Next to kininogens, which are inhibitors of thiol proteases, three other enzymes involved in thiol metabolism have also been detected, namely glutathione peroxidase 1, glutathione synthetase, and glutathione S-transferase P.

**Figure 3:**
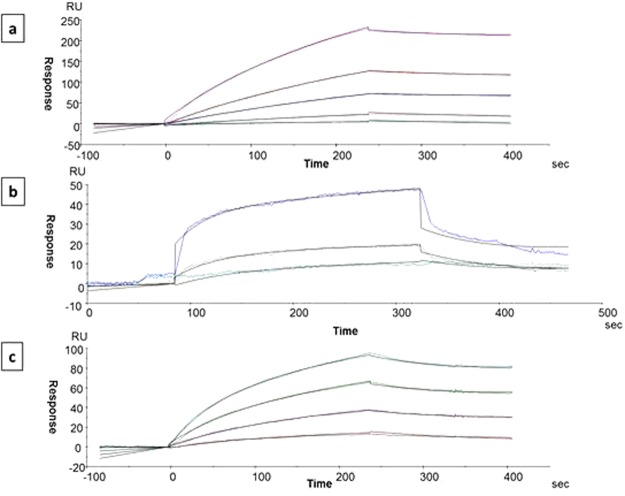
Interactions between polyclonal anti-ABCA1 antibody, evodiamine and ABCA1 protein (ABCA1 peptide from Abcam, #ab125995, covering C-terminal domain 2085–2245aa). **(a**) Interaction of polyclonal anti-ABCA1 antibody with immobilized ABCA1 on CM5 sensor chip as assessed by Biacore 3000. The interaction affinity is calculated with association constant (KA) equal to 1.18e6M and dissociation constant (KD) equal to 4.57e-7M (antibody concentrations from bottom to top are 4.25e-8, 8.5e-8,1.75e-7, 3.5e-7 and 7e-7M). **(b)** Interaction affinity between evodiamine and ABCA1. Evodiamine interacts with an association constant (KA) equal to 1,23e6M and a dissociation constant (KD) equal to 8,1e-7M (evodiamine concentrations from bottom to top are 2.5e-7, 5e-7 and 1e-6M). There is a bulk effect with Evodiamine at 1e-6M. This explains the higher signal (RU) in regard to the two lower concentrations. **(c)** Interaction of retinal dehydogenase-1 (ALDHA1) with immobilized ABCA1 peptide. The two proteins interact with a KA equal to 1.25e6M and KD of 8.03e-7M (ALDHA1 concentrations from bottom to top are; 6e-8, 1.25e-7, 2.5e-7, 5e-7M and 1e-6M).

ABCA1 is a central protein implicated in cholesterol transport. It may, thus, play an important role in mediating some of the reported positive cardiometabolic effects of evodiamine. To unambiguously confirm a direct interaction between ABCA1 and evodiamine, we performed surface plasmon resonance (SPR) analysis (Fig. 3). The quality of immobilization of recombinant ABCA1 peptide (Abcam #ab125995, covering C-terminal domain 2085–2245aa) was first assessed with a polyclonal anti-ABCA1 antibody (Fig. 3a). The interaction affinity was determined to be 4.58e-7M (KA = 2.18e61/M and KD = 4.58e-7M). SPR analysis revealed a direct interaction between ABCA1 and evodiamine, with a KD of 8.1e-7M (KA = 1.23e61/M) (Fig. 3b). As evodiamine is only soluble in organic solvents, a 1 mM evodiamine stock solution in DMSO was prepared. To avoid interference of the solvent with the SPR analysis, DMSO (10% v/v) was uniformly used and the optimal concentration range of evodiamine was determined to be between 2.5e-7M and 1e-6M. Among the recombinant proteins explored for their interaction ability i.e. ALDH1A1 (aldehyde dehydrogenase 1 family, member A1), natural human LMW (low molecular weight), and kinonogen (isoform 1 of kininogen-1), only ALDH1A1 could interact directly with immobilized ABCA1 with a KD equal to 8.03e-7M (KA = 1.25e61/M) (Fig. 3c).

### Evodiamine stabilizes ABCA1 and increases ChE

**Figure 4:**
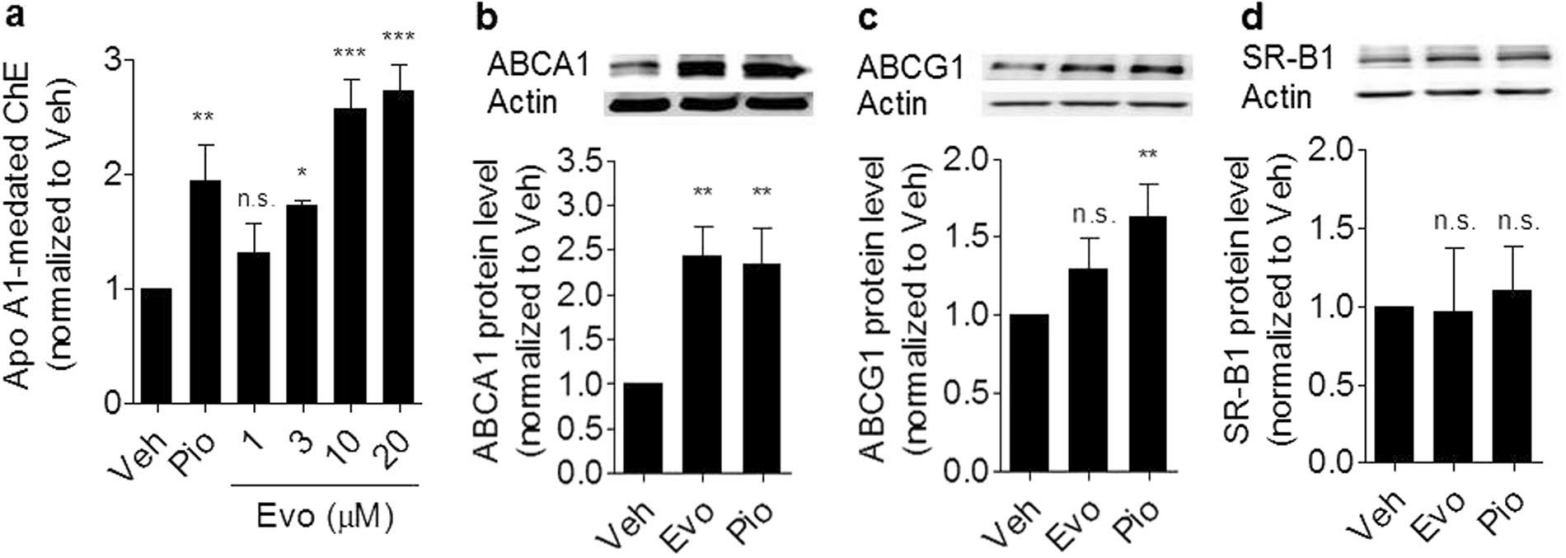
Evodiamine enhances apo A1-mediated ChE from THP-1 macrophages and increases ABCA1 protein level. (**a**) Differentiated THP-1 cells were loaded with [^3^H]-cholesterol together with the indicated treatments for 24 h. On the next day, the cells were washed twice with PBS and incubated with the same compounds [solvent vehicle control (Veh; ≤0.1% DMSO), evodiamine (1–20 μM), and the PPARγ agonist pioglitazone (10 μM) as positive control] with or without 10 µg/mL apo A1. Extracellular as well as intracellular radioactivities were quantified with scintillation counter. Differentiated THP-1-derived macrophages were treated with solvent vehicle control (Veh; ≤0.1% DMSO), evodiamine (10 μM), and the PPARγ agonist pioglitazone (10 μM) as positive control. After 24 h incubation, the cells were lysed and 20 μg protein was resolved via SDS-PAGE. Immunodetection was performed with antibodies against the indicated proteins, ABCA1 **(b)**, ABCG1 **(c)**, and SR-B1 **(d)**, and visualized by chemiluminescence detection. All experiments were performed at least three times and data are presented as means ± S.D. *vs*. solvent vehicle control, *p < 0.05, **p < 0.01, ***p < *0.001, n.s*. no significance (ANOVA/Bonferroni).

ABCA1 is a key player in RCT, since the transporter mediates ChE from peripheral cells. Intracellular free cholesterol (FC) transported through ABCA1 and subsequent binding to apo A1 is the predominant pathway for ChE. We, therefore, examined whether evodiamine binding to ABCA1 affects ChE from macrophages. In THP-1-derived macrophages, evodiamine concentration-dependently (1, 3, 10 and 20 µM) induced apo A1-mediated ChE above the level of the solvent vehicle control (Fig. 4a) comparably to the positive control pioglitazone, a selective PPARγ agonist^24^, known to increase cholesterol efflux and ABCA1 protein expression^25^. While evodiamine displayed a statistically significant effect on ChE when applied at a concentration as low as 3 µM (Fig. 4a), it did not affect THP-1 cell viability up to 40 µM (the highest concentration studied) as determined by quantifying cellular metabolic activity with resazurine conversion (Supplementary Fig. 1a).

Together with ABCA1, ABCG1 and SR-B1 are also relevant mediators of macrophage ChE^17,26,27^. Therefore, we tested the expression of these three transporter proteins upon evodiamine exposure in differentiated THP-1 macrophages with western blot analysis (Fig. 4b–d). The ABCA1 protein level was significantly upregulated by 10 μM evodiamine, similarly to the effect induced by the positive control pioglitazone applied at an equimolar concentration (Fig. 4b)^25^. However, no significant upregulation was observed for ABCG1 (Fig. 4c) and SR-B1 (Fig. 4d) in response to evodiamine. The levels of other membrane proteins, ABCA12, LDLR, and TFRC, were also not affected by the presence of 10 μM evodiamine (not shown).

**Figure 5:**
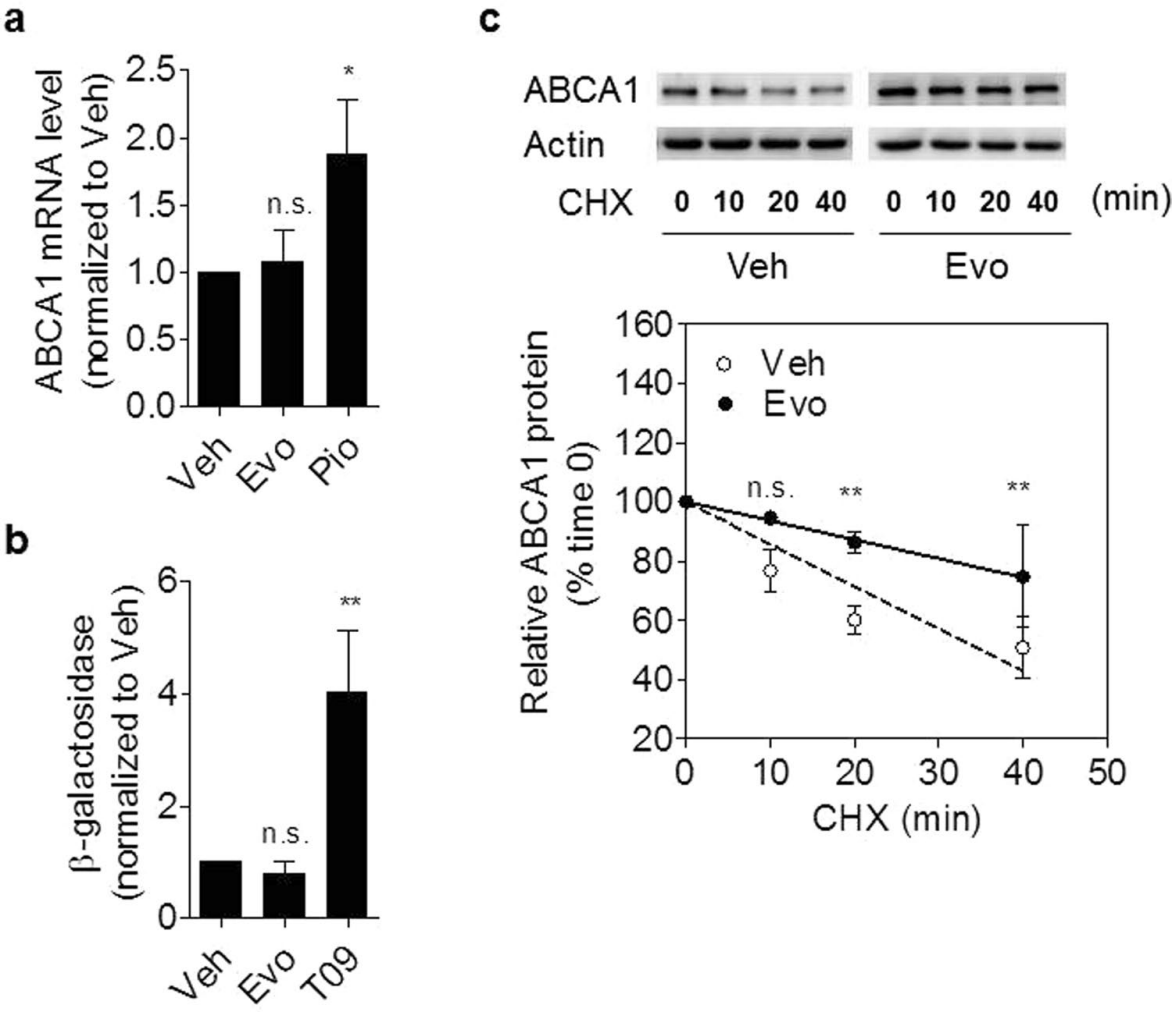
Effect of evodiamine on ABCA1 transcription and the degradation rate of ABCA1 protein. (**a**) Differentiated THP-1 macrophages were incubated with 10 μM evodiamine or 10 μM pioglitazone as positive control for 24 h. Total RNA was extracted and ABCA1 mRNA expression levels were quantified by qRT-PCR. **(b)** 293 T cells were transfected with a luciferase reporter construct driven by the human ABCA1 promoter as described in the Materials and Methods section. After transfection, cells were treated with 10 μM Evodiamine or 1 μM T0901317 as positive control for 24 h. **(c)** Differentiated THP-1 macrophages were incubated for 24 h with (black circle) or without (Veh; white circle) evodiamine (10 μM) and lysed after addition of cycloheximide (CHX; 140 μM) at different time points (0, 10, 20, 40 min). Western blot analysis shows the decline of ABCA1 protein level with cycloheximide in the presence and absence of evodiamine. All data are means ± S.D. (n = 3) *vs*. solvent vehicle control (DMSO), *p < 0.05, **p < *0.01, n.s*. no significance (ANOVA/Bonferroni).

One possibility to upregulate ABCA1 protein is *via* enhanced gene transcription leading to increased ABCA1 mRNA level^25,28^. We thus examined the effect of evodiamine on ABCA1 mRNA level by qRT-PCR in THP-1 macrophages (Fig. 5a). Consistent with former studies^25^, the positive control, pioglitazone induced ABCA1 mRNA, while evodiamine had no effect (Fig. 5a). In line with this finding, evodiamine also failed to enhance the expression of a luciferase reporter gene controlled by the human ABCA1 promoter (Fig. 5b).

### Evodiamine enhances ABCA1 protein half-life

Besides transcriptional upregulation, increased ABCA1 protein stability may lead to enhanced ABCA1 protein level^29^. Therefore, we studied ABCA1 protein half-life in the presence of evodiamine. Preliminary time course experiments revealed that evodiamine has a cumulative time-dependent effect on cellular ABCA1 protein levels, with a maximum (plateau level) after 24 h (Supplementary Fig. 1c). Thus, ABCA1 protein half-life was determined 24 h after treatment with evodiamine upon inhibition of *de novo* protein synthesis with cycloheximide (CHX, 140 μM). ABCA1 was stabilized in the presence of evodiamine, with a first significant difference to the vehicle control 20 min after the application of CHX (Fig. 5c). Forty minutes after CHX application, around 50% of ABCA1 was degraded in the presence of the solvent vehicle control, while the percentage of degradation was around 25% after evodiamine treatment, indicating an approximately two-fold slower ABCA1 degradation rate in the presence of evodiamine.

## Discussion

Using NPOT interactome analysis and SPR experiments, this study identifies ABCA1 as a direct binding target of evodiamine. Moreover, it demonstrates that evodiamine increases ChE from THP-1-derived macrophages and upregulates ABCA1 by protein stabilization.

NPOT is a novel label-free interactomics approach that provides significant advantages, since it does not require any conjugation or radioactive modifications of the used small molecule or the binding proteins. Furthermore, it is a relatively simple and fast approach with very few steps, i.e. lysate preparation, compound interaction in the NPOT system, isolation of heteroassemblies, and sequencing of the isolated heteroassembly peptides by LC/MS-MS. These features make NPOT a potentially optimal interactomics approach for target elucidation of pharmacologically active small molecules. To validate this technology for such application, we aimed to identify direct binding targets of the natural product evodiamine. As this compound is part of popular nutritional supplements, examining its biological activity is of very high relevance. NPOT (Fig. 2 and Supplementary Table 1) identified ABCA1 as a direct binding partner of evodiamine, and SPR (Fig. 3a,b) experiments indeed confirmed the indicated evodiamine-ABCA1 interaction. Interestingly, the NPOT (Fig. 2 and Supplementary Table 1) and SPR (Fig. 3c) experiments also revealed previously unknown direct protein-protein interaction between ABCA1 and ALDH1A1. A putative functional significance of this newly identified interaction remains to be investigated in future studies.

It is known that direct binding to ABCA1 protein could increase its stability^30^ and thermodynamic stabilization of proteins upon binding of small molecule ligands is a well-known phenomenon^31^. Therefore, we explored the potential of evodiamine to stabilize ABCA1 in intact cells and measured ChE as a functional readout of altered cellular ABCA1. Evodiamine treatment resulted in increased ChE from human macrophages. As an important part of the RCT, ChE is traditionally considered to counteract the development of atherosclerosis. However, recent studies in Ldlr^−/−^ mice lacking ABCA1 and ABCG1 in myeloid cells have indicated that anti-inflammatory effects of LXR activators are of key importance to their anti-atherosclerotic effects *in vivo*, independent of cholesterol efflux pathways mediated by macrophage ABCA1/G1^32^. Interestingly, previous studies reported an anti-atherosclerotic activity of evodiamine *in vivo*^33,34^. Therefore, it is tempting to speculate that this action could be explained at least in part by enhanced macrophage ChE. Indeed, increased macrophage ChE and ABCA1 upregulation in response to evodiamine was also reported in mouse macrophages, although pointing to a different mechanism of action implicating enhanced ABCA1 protein expression *via* a transcriptional effect^35^. This observed discrepancy might be due to different reasons (including, e.g., cell line-specific differences in the regulation, differences in the used culturing procedures, or maybe even potential species-specific regulation effects), and deserves to be further investigated in future studies.

ABCA1, ABCG1, and SR-B1 are the most important transporters contributing to ChE. ABCA1 is a ubiquitously expressed transporter protein and promotes ChE predominantly to lipid poor apoA1. Loss of ABCA1 is the molecular basis of Tangier disease associated with low level of apoA1 and accumulation of cholesterol in macrophages^18^. ABCG1 is also known to be expressed in macrophages and promotes ChE from macrophages binding to HDL^36^. Double knockdown of ABCA1 and ABCG1 in mice decreases ChE *in vitro* and RCT *in vivo* stronger when compared with single knockdown, indicating a cooperative additive effect of these two transporters^37^. SR-B1, the first well-defined HDL receptor, plays a significant role in promoting cholesterol uptake in liver cells, and therefore promotes RCT *in vivo*^38–40^. It is also reported that SR-B1 mediates ChE in other cell types, e.g. CHO cells^41^ and probably its action is dependent on interactions with lipid-bound apoE^42^. In our study, evodiamine up-regulated ABCA1 significantly without altering the expression level of ABCG1 or SR-B1 proteins, implying a ChE-enhancing mechanism involving specifically the ABCA1 transporter.

To exclude a potential transcriptional influence of evodiamine on ABCA1 expression, ABCA1 mRNA was quantified by qRT-PCR. In contrast to pioglitazone, ABCA1 mRNA level remained unaffected in the presence of evodiamine. Moreover, evodiamine also failed to induce transactivation of a human ABCA1 promoter luciferase reporter construct, overall implying a mechanism of action different from the transcriptional upregulation induced by the PPARγ agonist pioglitazone. Noteworthy, although we have used a PPARγ agonist as a positive control in most of the experiments, in general LXR ligands are more suitable and more widely used as a positive control for activation of ABCA1 expression and macrophage cholesterol efflux (e.g., the LXR ligand T0901317, which we used as a positive control for the experiment presented on Fig. 5b). Increased protein level might be in general due to an increased rate of protein synthesis or due to an increased protein half-life. Stabilization of proteins upon binding of small molecule ligands is a well-known phenomenon^31^, and previous studies reveal that direct binding to ABCA1 protein could increase its stability^30^. Indeed, quantification of the ABCA1 protein half-life revealed that it was approximately doubled in the presence of evodiamine **(**Fig. 4c**)**.

In conclusion, we validate the application of NPOT as a highly promising interactomics approach to identify molecular targets of small molecules. Furthermore, we report for the first time that the natural product evodiamine binds to ABCA1 and increases its protein stability, resulting in elevated cellular ABCA1 and increased ChE from human THP-1-derived macrophages. This mechanism of action may contribute to the previously described anti-atherosclerotic effect of evodiamine, and lead to a better understanding of the biological effects induced by dietary supplements containing this natural product.

## Methods

### Reagents

Evodiamine (#E3531), phorbol 12-myristate 13-acetate (PMA) (#P1585), water soluble cholesterol (#C4951), ApoA1 (#73366), cycloheximide (#C7698), T0901317 (#T2320), and digitonin (#D141) were purchased from Sigma-Aldrich (Vienna, Austria), and pioglitazone (#M35102242) was obtained from Molekula (Munich, Germany). [^3^H]-cholesterol (#NET139001MC; 1 mCi, 37 MBq) was provided by Perkin Elmer Life Sciences (Vienna, Austria). The tested compounds were dissolved in DMSO, aliquoted and stored at −20 °C until use. An equal amount of DMSO was always tested in each condition in all experiments to assure that the solvent vehicle does not influence the results itself. Primary antibodies against ABCA1 (#NB400-105), ABCG1 (#NB400-132), ABCA12 (NB100-93466) and SR-B1 (#NB400-104) were obtained from Novus Biologicals (Vienna, Austria). The anti-actin antibody (#8691002) was acquired from MP biologicals (Illkirch, France). Primary antibodies against TRFC (#12113) and LDLR (#SC-18823) were bought from Cell Signaling (Austria) and Santa Cruz (Austria), respectively. HRP-linked anti-rabbit IgG secondary antibody (#7074S) was purchased from New England Biolabs (UK), and horseradish peroxidase conjugated goat anti-mouse secondary antibody (#12-349) from Upstate (Millipore, Vienna, Austria). All antibodies were used in a dilution of 1:500.

### Cell culture

Human THP-1 monocytic cells (ATCC) were cultivated in RPMI-1640 medium (Lonza, Basel, Switzerland) supplemented with 10% fetal bovine serum (FBS) (Gibco, Lofer, Austria), 1% L-glutamine, 100 U/mL penicillin and 100 µg/mL streptomycin, at 37 °C and 5% CO_2_. The THP-1 cells were differentiated into macrophages upon stimulation with 200 nM PMA for 72 h^25,43^.

HEK-293T cells were cultured in phenol red-free DMEM medium (Life Technologies, Grand Island, New York) supplemented with 10% (v/v) FBS (HyClone, Logan, Utah), 2 mM L-glutamine, 100 U/mL penicillin, 100 μg/mL streptomycin, 0.25 μg/mL amphotericin, and 1 mM sodium pyruvate, at 37 °C and 6% CO_2_ in a humidified atmosphere.

### Nematic protein organisation technique (NPOT), heteroassemblies isolation, and proteomics

The NPOT proprietary technology is based on the Kirkwood-Buff molecular crowding and aggregation theory^15,16^. THP-1 cell lysates were prepared under low temperature (4 °C) in the absence of any detergent, reducing agent or protease or phosphatase inhibitors. All probable dilutions and washes are performed in HBSS with equal osmolality, trace elements, vitamins and salts in concentrations as close as possible to those of the interstitial medium or cytoplasm. Evodiamine (10^−6^M) is put in contact with the total tissue material. The macromolecular assemblies related to the ligand are separated using a differential microdialysis system, based on a transitory pH gradient (5–10) wherein the macromolecules (protein groups) migrate in the liquid phase based on their physico-chemical properties. The use of pH gradient in NPOT is important in order to cover the range of existing physiological extracellular and intracellular pH values, to assure the detection of relevant interactions which might be pH-dependent. The migrating macromolecules growing gradually form nematic crystals to macromolecular heteroassemblies thanks to the molecular interactions between evodiamine and its targets. The heteroassemblies are trapped in mineral oil. The latter are isolated and identified by mass spectroscopy directly in liquid.

Under a Microscope, each heteroassembly formed was isolated by microdissection and washed in acetone prior to solubilisation in standard Hepes saline buffer solution (HBSS solution).

Prior to LC-MS/MS experiments heteroassemblies were solubilised directly in 10 µl of 2D buffer (7M Urea, 2M Thiourea, 4% CHAPS, 20 mM DTT, 1 mM PMSF). Proteins were precipitated in acetate buffer by centrifugation for 20 min at 7500xg. Thereafter pellets were digested for 1 h with trypsin Gold (Promega) at 37 °C. Trypsin Gold was resuspended at 1 µg/µL in 50 mM acetic acid, and then diluted in 40 mM NH_4_HCO_3_ to 20 µg/ml. The samples were dried in SpeedVac^®^ at room temperature. Peptides were purified and concentrated by using ZipTip^®^ pipette tips (Millipore Corporation) before proceeding for mass spectrometry analysis through 1 h LC-MS/MS analyses protocol in an ESI-QUAD-TOF machine. Proteins were identified using Mascot software.

### Surface Plasmon Resonance (SPR), Biacore 3000

Prior to protein immobilization all proteins were dialysed against HBS-E (10 mM Hepes, 150 mM NaCl, 3 mM EDTA) buffer in order to eliminate the TRIS that would interact with the primary amine on the surface of the CM5 chip. Accordingly, 100 ml ice cold HBS-E buffer filtered through a 0.45 µM membrane was prepared. Recombinant proteins were slowly defrosted on ice and transferred to the dialyse slide according to the manufacturer instruction. Slides were inflated in 100 ml ice cold HBS-E buffer and dialysed for 2 h, and then the buffer was changed and followed by dialysis over night at 4 °C using a magnetic stirrer. Recombinant proteins (ABCA1 peptide (Abcam #ab125995, covering C-terminal domain 2085–2245aa), regucalcine, natural human LMW kinonogen, and retinal dehydrogenase −1 (ALDH1A1)) were immobilised on CM5 (carboxymethylated dextran covalently attached to a gold surface) using the amine coupling method according to manufacturer’s recommendation. Briefly the CM5 surface was activated with the mixture of EDC/NHS. Recombinant proteins at dilution 1 µg/ml in a corresponding buffer *i.e*. ABCA1 and retinal dehydrogenase-1 in sodium acetate buffer 100 mM and regucalcine as well as natural human LMW kinonogen in formate buffer 100 mM. The control channel was activated with EDC/NHS. All channels were thereafter saturated with 0.1M ethanolamine solution.

Evodiamin was first solubilised in DMSO (dimethyl sulfoxide) at stock solution 1 mM. It was thereafter diluted in HBS-E, centrifuged at 2500 × g for 10 min to roll out the presence of non-solubilised compound. Evodiamine at concentration 2.5e-7, 5e-7 and 1e-6M was injected over the four flow cells at a flow rate equal to 30 µl/h, with KINJET 60/180%pos, analyte position, volume and dissociation time respectively. The sensor chip surface was regenerated with 1 mM HCl. Polyclonal anti-ABCA1 antibody at concentrations 4.25e-8, 8.5e-8, 1.75e-7, 3.5e-7 and 7e-7M or recombinant human retinal hydogenase-1 (ALDHA1) at concentrations 6e-8,1.2e-7, 2.5e-7, 5e-7 and 1e-6M were also injected over the four flow cells at a flow rate equal to 30 µl/h, with KINJET 60/180%pos, analyte position, volume and dissociation time respectively. The specific binding was calculated using Biaeval 3 software (Biacore, GE Healthcare) with the postulate of 1:1 ligand/analyte interaction. Based on the Chi2 value in regard to the Rmax (less than 10%) and the difference interval ±2 RU theoretic fitting and experimental results best fitting algorithm between Langmuir or drifting base line was applied.

### Cell viability evaluation

Fluorescent resazurin assay was used to determine cell viability as described^44^. The weakly fluorescent blue resazurin is irreversibly reduced to the pink colored and highly red fluorescent resorufin in living cells, which makes it a useful indicator for cell viability determination. In this assay, THP-1 cells were seeded at a density of 0.2 × 10^6^ cells/mL in 100 μL/well RPMI-1640 medium supplemented with 10% FBS, 1% L-glutamine and 200 nM PMA in 96-well microtiter plates to allow differentiation into adherent macrophage-like cells. After 72 h, the medium was replaced with FBS-free RPMI-1640 medium supplemented with 0.1% BSA and 20 µg/mL unesterified cholesterol and cells were treated with solvent vehicle control (DMSO), 50 μg/mL digitonin as a positive control, or varying concentrations of evodiamine. After 24 h, cells were washed with pre-warmed PBS and incubated for 4 h with PBS containing 10 μg/mL freshly prepared resazurin. The assay was performed in triplicate and the fluorescence was determined at 580 nm-emission/535 nm-excitation with a Tecan GENiosPro plate reader (Männedorf, Switzerland).

### Cholesterol efflux assay

The assay was performed in 24-well plates with THP-1 monocytes seeded at a density of 0.2 × 10^6^ cells/mL. After differentiation with PMA for 72 h, THP-1 macrophages were washed once with PBS and incubated with DMSO, pioglitazone (as positive control) and evodiamine at indicated concentrations together with [^3^H]-cholesterol (0.2 µCi/ml; #NET139001MC, Perkin Elmer) and unlabeled cholesterol (20 µg/ml; #C4951, Sigma-Aldrich) in FBS-free RPMI-1640 medium containing 0.1% BSA. After equilibrating the cholesterol pools for 24 h, cells were washed twice with PBS and incubated in FBS-free RPMI-1640 medium with the same compounds (DMSO, pioglitazone, or evodiamine) in the absence or presence of apoA1 (10 µg/mL) or human plasma (1%) for 6 h. The [^3^H]-cholesterol released into the medium and the total cell-associated radioactivity (cell lysates) were quantified by liquid scintillation counting. The assay was performed in triplicate and the percentage of apoA1- or human plasma-mediated cholesterol efflux was calculated according to the following equations^43^:

1 $$\begin{array}{c}Apo\,A1\,mediated\,cholesterol\,efflux\, \% \\ \,\,\,=\,(\frac{(extracellular\,cpm)\,apo\,A1}{(total\,cpm)\,apo\,A1}-\frac{(extracellular\,cpm)\,no\,apo\,A1}{(total\,cpm)\,no\,apo\,A1})\times 100\end{array}$$

2 $$\begin{array}{c}Human\,plasma\,mediated\,cholesterol\,efflux\, \% \\ \,\,\,=\,(\frac{(extracellular\,cpm)\,plasma}{(total\,cpm)\,plasma}-\frac{(extracellular\,cpm)\,no\,plasma}{(total\,cpm)\,no\,plasma})\times 100\end{array}$$

### Transfections, luciferase, and β-galactosidase assays

The luciferase and β-galactosidase assays were performed as previously described. Briefly, 293 T cells were seeded in a 24-well plate at a density of 0.05 × 10^6^ cells/mL and 500 μL medium per well. 24 h later, cells were transfected with a reporter plasmid containing the human ABCA1 promoter cloned in pGL3-basic (0.33 μg of plasmid/well) using FuGENE 6 (Promega) according to the manufacturer’s protocol (3:1 FuGENE 6:DNA ratio). A β-galactosidase expression plasmid (0.33 μg/well) was used to normalize for transfection efficiency. Upon transfection, cells were further incubated under normal growth conditions or treated either with the synthetic LXR ligand T0901317 (final concentration 1 μM; used as a positive control) or evodiamine (final concentration 10 μM) for 24 h. Each transfection reaction was carried out in triplicate and luciferase activity was measured and normalized to β-galactosidase activity.

### Protein extraction and western blotting

Differentiated THP-1 macrophages were treated with solvent vehicle control (DMSO), evodiamine, or pioglitazone at the indicated concentrations as stated in the respective figure legends. After 24 h incubation, cells were lysed using NP40 lysis buffer (150 mM NaCl; 50 mM HEPES (pH 7.4); 1% NP40; 1% protease inhibitor Complete^TM^ (Roche); 1% phenylmethylsulfonyl fluoride (PMSF); 0.5% Na_3_VO_4_; 0.5% NaF) for 30 min on ice. Total protein in lysates was quantified by the bradford assay (Roti^®^-Quant, #K015.1, Carl Roth) and 20 μg protein was loaded per lane. The proteins were resolved via SDS-PAGE and subjected to immunoblotting. Blots were visualized using the ECL reagent and an LAS-3000 luminescent image analyzer (Fujifilm) with AIDA image analyser 4.06 software (Raytest).

### qRT-PCR

For quantitative reverse transcription PCR analysis (qRT-PCR), cells were treated with solvent vehicle control (DMSO), evodiamine, and pioglitazone at the indicated concentrations. After 24 h, total RNA was extracted from cells using the peqGOLD Total RNA Kit (PeqLab, Linz, Austria). 1 μg RNA was used for cDNA synthesis with oligo (dT) and MultiScribe Reverse Transcriptase (Applied Biosystems). 40 ng cDNA per well was used for PCR amplification with the primers for ABCA1 (HS_ABCA1_1_SG QuantiTect primer assay, Cat.no.: #QT00064869, Qiagen) and the LightCycler 480 SYBR Green I Master kit (Roche) with a LightCycler 480 (Roche). Each sample was run in triplicate and the results were analyzed according to the ddCt method. 18S rRNA (Hs_RRN18S_1_SG QuantiTect Primer assay, Cat.no.: #QT00199367, QIAGEN) was used as a reference gene.

### Data analysis and statistics

Results are presented as means ± SD from at least three independent experiments. One-way analysis of variance (ANOVA) was performed for comparison of different treatment conditions. GraphPad Prism software (version 4.03) was used for statistical analysis (GraphPad Software Inc., La Jolla, CA). *P* < *0.05* is considered to be significant (*p < 0.05, **p < 0.01, ***p < *0.001*).

## Electronic supplementary material

Supplementary information
