# Supplementary material for: Novel interactomics approach identifies ABCA1 as direct target of evodiamine, which increases macrophage cholesterol efflux
Source: Sci Rep. 2018 Jul 23;8:11061. doi: 10.1038/s41598-018-29281-1 (PMC6056500; doi:10.1038/s41598-018-29281-1)
Supplement: Supplementary file 1 — Supplementary information [file 41598_2018_29281_MOESM1_ESM.docx]

**Supplementary information**

**Novel interactomics approach identifies ABCA1 as direct target of evodiamine, which increases macrophage cholesterol efflux**

Limei Wang^1, 2^, Pierre Eftekhari^3^, Daniel Schachner^1^, Irena D. Ignatova^4^, Veronika Palme^1^, Nicole Schilcher^1^, Angela Ladurner^1^, Elke H. Heiss^1^, Herbert Stangl^5^, Verena M. Dirsch^1^, Atanas G. Atanasov^1,6^*

*^1^Department of Pharmacognosy, University of Vienna, Vienna, Austria*

*^2^Department of Pharmacology, School of Pharmacy, Qingdao University, Qingdao 266021, Shandong Province, China*

*^3^Inoviem Scientific, Strasbourg, France*

*^4^Department of Pharmacology, University of Virginia Health System, Charlottesville, VA, USA*

*^5^Institute of Medical Chemistry, Center for Pathobiochemistry and Genetics, Medical University of Vienna, Vienna, Austria*

*^6^* *Institute of Genetics and Animal Breeding of the Polish Academy of Sciences, 05-552 Jastrzebiec, Poland*

**Keywords:** Evodiamine, Cholesterol efflux, ABCA1, Interactome analysis, Macrophages

**Correspondence:** Dr. Atanas G. Atanasov, Department of Pharmacognosy, Faculty of Life Sciences, University of Vienna, Althanstrasse 14, A-1090 Vienna, Austria

**E-mail:** [atanas.atanasov@univie.ac.at](mailto:atanas.atanasov@univie.ac.at)

**Supplementary Figure 1 │Evodiamine enhances human plasma-mediated ChE and increases ABCA1 protein expression in a time dependent manner.** **(a)** Differentiated THP-1 macrophages were incubated with solvent vehicle control (DMSO), 50 μg/mL digitonin, and indicated concentrations of evodiamine. After 24 h, the increased fluorescent signal was detected as a measure for cell viability. **(b)** Differentiated THP-1 cells were loaded with [^3^H]-cholesterol together with the indicated treatments for 24 h. On the next day, the cells were washed twice with PBS and incubated with the same compounds [solvent vehicle control (Veh; ≤ 0.1% DMSO), evodiamine (1-20 μM), and the PPARγ agonist pioglitazone (10 μM) as positive control] with or without 1% human plasma with 10 μM evodiamine dissolved in serum-free medium for 6 h. Extracellular as well as intracellular radioactivity were quantified with scintillation counter. **(c)** Differentiated THP-1 macrophages were incubated up to 24 h with (black circles) or without (Veh; white circles) evodiamine (10 μM). Cells were lysed at different time points (0, 3, 6, 15, 24 h) and 20 μg protein was resolved via SDS-PAGE. Immunodetection was performed with the antibody against ABCA1. All data are means ± S.D. (n=3) vs. solvent vehicle control (DMSO) at certain time point, **p <0.05, ***p <0.001, n.s.* no significance (ANOVA / Bonferroni).

**Supplementary Table 1│**Interactions of proteins with evodiamine identified by the NPOT analysis. Total of 26 proteins are identified through the LC/MS-MS analysis. Among these proteins 14 have been implicated in atherosclerosis (highlighted proteins in bold).

| **Accession No.** | **Identified protein** | **Peptide Sequence** | **Coverage (%)** | **MW** |
| --- | --- | --- | --- | --- |
| **Q06278** | **Aldehyde oxidase** | **QENALAIVNSGMR** | **1** | **147917** |
| **P01019** | **Angiotensinogen** | **VANPLSTA** | **2** | **53154** |
| **P10644** | **cAMP-dependent protein kinase type I-alpha regulatory subunit** | **SENEEFVEVGR** | **3** | **42981** |
| **O95477** | **ATP-binding cassette sub-family A member 1** | **VSELCGLPREKLAAA-QEMDLVRMLL-NLNKLEPIATEVWLINKSMEL** | **2** | **254301** |
| **P01024** | **Complement C3** | **SNLDEDIIAEENIVSR-TELRPGETLNVNFLLR-TMQALPYSTVGNSNNYLHLSVLR-IPIEDGSGEVVLSR-TIYTPGSTVLYR** | **5** | **187148** |
| **P0C0L4** | **Complement C4-A** | **QGSFQGGFR-VTASDPLDTLGSEGALSPGGVASLLR-VGDTLNLNLR-TTNIQGINLLFSSR-TTNIQGINLLFSSRR** | **3** | **192785** |
| **P02748** | **Complement component C9** | **VVEESELAR** | **2** | **63173** |
| **P00450** | **Ceruloplasmin** | **QSEDSTFYLGER** | **1** | **122205** |
| **P00488** | **Coagulation factor XIII A chain** | **AVPPNNSNAAEDDLPTVELQGVVPR** | **3** | **83267** |
| O75891 | Cytosolic 10-formyltetrahydrofolate dehydrogenase | FADGDLDAVLSR | 1 | 98829 |
| P38117 | Electron transfer flavoprotein subunit beta | EIDGGLETLR-LGPLQVAR | 7 | 27843 |
| P30043 | Flavin reductase (NADPH) | NDLSPTTVMSEGAR | 7 | 22119 |
| **P02675** | **Fibrinogen beta chain** | **QDGSVDFGR** | **2** | **55928** |
| **P02679** | **Fibrinogen gamma chain** | **VELEDWNGR** | **2** | **51511** |
| P07203 | Glutathione peroxidase 1 | DYTQMNELQR | 5 | 21938 |
| P48637 | Glutathione synthetase | QYSLQNWEAR-AIENELLAR | 4 | 52384 |
| P09211 | Glutathione S-transferase P | PPYTVVYFPVR | 5 | 23355 |
| Q9BSH5 | Haloacid dehalogenase-like hydrolase domain-containing protein 3 | IFQEALR-LAVISNFDR | 6 | 27999 |
| P69905 | Hemoglobin subunit alpha | MFLSFPTTK-MFLSFPTTK-VGAHAGEYGAEALER | 17 | 15257 |
| P02042 | Hemoglobin subunit delta | LLGNVLVCVLAR-LLVVYPWTQR-VNVDAVGGEALGR | 24 | 16055 |
| **P01042** | **Kininogen-1** | **QVVAGLNFR** | **1** | **71957** |
| **Q15493** | **Regucalcin** | **YFAGTMAEETAPAVLER-VTMDAPVSSVALR** | **10** | **33252** |
| P09455 | Retinol-binding protein 1 | ALDVNVALR | 7 | 15850 |
| P00352 | Retinal dehydrogenase 1 | IFVEESIYDEFVR-IFVEESIYDEFVRR-TIPIDGNFFTYTR-QAFQIGSPWR | 7 | 54861 |
| **Q08257** | **Quinone oxidoreductase** | **GTIEINPR** | **2** | **35206** |
| P10809 | 60 kDa heat shock protein, mitochondrial | VTDALNATR | 2 | 61054 |
